# Different Paths, Same Goals: A Comparative Study on the Safety of Femoral vs. Axillary Arterial Cannulation in VA ECMO

**DOI:** 10.3390/jcm14134613

**Published:** 2025-06-29

**Authors:** Tahsin Murat Tellioglu, Hasan Iner, Erturk Karaagac, Muhammed Cagri Yalcin, Mustafa Gurbuz, Yuksel Besir, Orhan Gokalp, Levent Yilik

**Affiliations:** 1 Department of Cardiovascular Surgery, Hatay Training and Research Hospital, Antakya 31027, Hatay, Türkiye; 2 Department of Cardiovascular Surgery, Ataturk Training and Research Hospital, Izmir Katip Celebi University, Karabaglar 35360, Izmir, Türkiye; hasan_iner@hotmail.com (H.I.); erturkkaraagac@gmail.com (E.K.); yukselbesir@gmail.com (Y.B.); gokalporhan@yahoo.com (O.G.); levent.yilik@ikc.edu.tr (L.Y.); 3 Department of Cardiovascular Surgery, Izmir Ataturk Training and Research Hospital, Karabaglar 35360, Izmir, Türkiye; dr.cagriyalcin@gmail.com; 4 Department of Cardiovascular Surgery, Graduate School of Health Sciences, Izmir Katip Celebi University, Cigli 35620, Izmir, Türkiye; mustafagurbuz91@yahoo.com

**Keywords:** VA ECMO, cardiogenic shock, femoral cannulation, axillary cannulation, cannulation complications, extracorporeal life support, vascular access

## Abstract

**Objectives:** This study aimed to evaluate the impact of cannulation site preference—femoral versus axillary—on postoperative complications and in-hospital mortality in patients undergoing peripheral venoarterial extracorporeal membrane oxygenation (VA ECMO) due to cardiogenic shock. **Methods:** In this single-center, retrospective study, 85 patients who received peripheral VA ECMO support between January 2013 and July 2023 were analyzed. Patients were divided into two groups based on arterial cannulation site: femoral cannulation (FC, n = 47) and axillary cannulation (AC, n = 38). Preoperative, intraoperative, and postoperative variables were compared. Cannulation-related complications were categorized as vascular, neurological, or pulmonary. The primary endpoints were postoperative complications and in-hospital mortality. **Results:** There were no statistically significant differences between the FC and AC groups in terms of demographics, comorbidities, surgical procedures, or ECMO weaning times. Rates of vascular, neurological, and pulmonary complications were similar between groups. Mortality and postoperative dialysis rates did not differ significantly. The low rate of ischemic complications in the FC group may be explained by the use of distal perfusion catheters, which are considered the standard approach to prevent leg ischemia. Both cannulation techniques demonstrated comparable safety and efficacy profiles. **Conclusions:** Both femoral and axillary cannulation sites can be safely used for peripheral VA ECMO when selected based on individual patient conditions and institutional experience. Cannulation strategy should be tailored according to the urgency of the clinical situation, anatomical feasibility, and anticipated duration of support. Further prospective, randomized studies are required to establish the optimal cannulation approach.

## 1. Introduction

Cardiogenic shock is a severe clinical condition characterized by systemic hypoperfusion and impaired myocardial function, associated with a high mortality rate [1,2]. It can result from various causes, including acute myocardial infarction, myocarditis, trauma, sepsis, decompensated heart failure, and post-cardiotomy conditions. Particularly, post-cardiotomy cardiogenic shock, observed in patients who cannot be stabilized despite intensive inotropic support and intra-aortic balloon pump (IABP) therapy, is a rare but high-risk condition [3]. When conventional treatment methods prove insufficient, mechanical circulatory support systems play a critical role [4].

Veno-arterial extracorporeal membrane oxygenation (VA ECMO) is a widely used and reliable temporary support therapy for the treatment of cardiogenic shock. However, despite technological advancements and increasing clinical experience, serious complications can still arise during VA ECMO, either due to the procedure itself or the patient’s critical condition [5]. These complications may be related to cannulation preferences.

Peripheral cannulation is the most common method for adult VA ECMO, with arterial access through the femoral or axillary arteries. The femoral approach is quick and simple, suitable for emergencies but carries higher risks of limb ischemia and vascular injury. Axillary cannulation offers better mobility and blood flow, with a lower risk of ischemia, but is surgically more complex.

Current evidence comparing femoral and axillary cannulation strategies in VA ECMO remains limited and inconclusive. Some studies suggest that axillary access may reduce limb-related complications and improve upper-body perfusion [6,7]. In contrast, other reports have not demonstrated significant differences in major outcomes such as survival or neurological complications [8,9]. Further high-quality prospective studies are warranted to establish best practices.

It is known that vascular, neurological, and pulmonary complications related to cannulation preferences can limit the benefits provided by ECMO [5]. However, the specific impact of these complications on mortality has not been clearly demonstrated. Moreover, there are very few studies that have thoroughly examined the effects of cannulation preference on perioperative survival and postoperative complications. This study aims to evaluate the impact of cannulation preference on postoperative complications and mortality in patients with cardiogenic shock who underwent VA ECMO with peripheral cannulation. The data obtained are expected to contribute to clinical practice in terms of patient selection, cannulation strategies, and complication prevention approaches in ECMO management.

## 2. Patients and Methods

In our single-center, retrospective study, 85 adult patients (25 female, 60 male) who underwent peripheral VA ECMO due to cardiogenic shock (CS), despite conventional treatment or IABP therapy while being monitored in intensive care between January 2013 and July 2023, were evaluated. CS was defined as a cardiac disorder leading to a systolic blood pressure <90 mm Hg for ≥30 min (or the need for vasopressors, inotropes, or mechanical circulatory support (MCS) to maintain systolic blood pressure ≥90 mm Hg with evidence of tissue hypoperfusion) [10].

All patients were divided into two groups according to their arterial cannulation preferences: femoral (FC) (n:47) and axillary (AC) (n:38). Clinical data were retrieved from the hospital information management system (HIMS), medical archive records and epicrisis reports. The patients were analyzed based on preoperative, perioperative, and postoperative outcomes. The study was approved by the İzmir Katip Çelebi University Faculty of Medicine Local Ethics Committee (Decision Date: 15 June 2023, IRB: 0304).

### 2.1. Study Variables and Endpoints

Preoperative data for ECMO included age, sex, body surface area (BSA), diabetes mellitus (DM), hypertension (HT), hyperlipidemia (HLP), chronic obstructive pulmonary disease (COPD), peripheral artery disease (PAD), chronic kidney disease (CKD), history of myocardial infarction (MI), Euroscore II, smoking status, and IABP use.

Intraoperative data consisted of the surgical procedure, total cardiopulmonary bypass (CPB) time, cross-clamp (CC) time, and ECMO timing.

Postoperative data included mechanical ventilation duration, the amount of blood products used, presence of neurological complications, need for hemodialysis, drainage volume, intensive care unit (ICU) stay, hospital stay, in-hospital mortality, and ECMO weaning times.

Postoperative cannulation-related complications and mortality after peripheral VA ECMO application were planned as the primary endpoints. Secondary endpoints: Mechanical ventilation time, blood product use, need for hemodialysis, ICU and hospital length of stay, and ECMO weaning time. All outcomes were assessed during hospitalization.

### 2.2. Cannulation Preference and Surgical Technique

All peripheral VA ECMO procedures were performed by the same surgical and anesthesia teams either in the operating room or the cardiovascular ICU. If the patient was in the ICU and the procedure was urgent, femoral cannulation was preferred either in the ICU or operating room. If the patient was in the ICU and the procedure was not urgent, VA ECMO was applied via axillary cannulation in the operating room.

Femoral Cannulation (FC) was performed percutaneously using the Seldinger technique in the operating room or ICU. The femoral artery was cannulated using a 19-23Fr arterial cannula (Maquet HLS, Getinge Group, Rastatt, Germany), and the femoral vein was cannulated with a 21-25Fr two-stage venous cannula (Maquet HLS, Getinge Group, Rastatt, Germany). The two-stage venous cannula refers to a design with two drainage ports, typically used to improve venous drainage. These cannulas are approved for extended extracorporeal life support (ECLS) use. A distal perfusion catheter (6-8Fr) was routinely placed anterogradely distal to the arterial cannula to ensure extremity perfusion in patients undergoing femoral artery cannulation.

Axillary Cannulation (AC) was performed under general anesthesia in the operating room. The right subclavian artery was exposed. After systemic heparinization, clamps were applied proximally and distally on the artery, and arterial cannulation was performed via a 6–8–10 mm Dacron graft using a 19-23Fr arterial cannula (Maquet HLS, Getinge Group, Rastatt, Germany). The femoral vein was cannulated percutaneously using a 21-25Fr two-stage venous cannula (Maquet HLS, Getinge Group, Rastatt, Germany). Cannulation was performed using the Seldinger technique.

Cannulation-related complications were classified as vascular, neurological, and pulmonary complications. Vascular complications were defined as hemorrhage or hematoma requiring surgical intervention, and extremity ischemia that required surgical intervention (fasciotomy, amputation, etc.). Neurological complications were defined as seizures, encephalopathy, intracerebral hemorrhage, and ischemic stroke in the early postoperative period. Pulmonary complications were defined as pneumonia, pulmonary congestion, and the need for tracheostomy. Mortality was defined as in-hospital mortality.

### 2.3. Management and Monitoring of VA ECMO Patients

In all patients undergoing VA ECMO, the ECMO blood flow was gradually increased to reach the optimal level following cannulation (approximately 4–5 L/min [~80% of cardiac output] in left or biventricular failure, and 3–5 L/min [~60–80% of cardiac output] in isolated right ventricular failure). Once respiratory and hemodynamic targets were achieved, the flow was maintained at that level. Continuous monitoring included cardiac rhythm, oxygen saturation, invasive arterial blood pressure, and central venous pressure (CVP). VA-ECMO management and weaning were performed according to the criteria proposed by the Extracorporeal Life Support Organization guidelines.

Anticoagulation was initiated with an initial dose of 75 units/kg of heparin. The target activated clotting time (ACT) was maintained between 180–220 s, and the activated partial thromboplastin time (aPTT) was targeted at 1.5 times the normal range. In cases of heparin-induced thrombocytopenia (HIT) or when long-term ECMO support was anticipated, direct thrombin inhibitors such as bivalirudin were used as alternatives to heparin. Anticoagulation was temporarily discontinued in life-threatening bleeding events or when additional surgical procedures were required.

The ECMO circuit, including the oxygenator and tubing, was carefully inspected during clinical assessments performed by the ECMO team at the bedside twice daily. These evaluations aimed to detect indirect signs of insufficient anticoagulation, such as fibrin deposits or thrombus formation. In included patients, extubation was planned in suitable cases to prevent lung injury due to barotrauma or high-concentration oxygen exposure. Otherwise, early tracheostomy was considered.

All patients were frequently assessed using transthoracic echocardiography (TTE) to evaluate volume status, cardiac contractility, thrombus presence, and aortic valve opening. If clinically indicated, an IABP was added to the VA ECMO system to assist in left ventricular decompression.

Continuous renal replacement therapy (CRRT) was initiated in patients with persistent renal dysfunction and oliguria/anuria unresponsive to diuretic therapy after 48 h. Daily physical assessments included peripheral pulse examination (via palpation and Doppler ultrasound), neurological evaluation, and respiratory examination. Cannulation sites were observed daily for signs of hematoma, bleeding, or wound infection.

Weaning from VA ECMO was considered when pulmonary and hemodynamic recovery had been achieved. The weaning process was conducted in accordance with the criteria recommended by the Extracorporeal Life Support Organization (ELSO) guidelines. Both pulmonary and hemodynamic improvement and stabilization were required for weaning attempts. Indicators of cardiac recovery included increased blood pressure without inotropic support, the return of pulsatile arterial waveforms, a drop in PaO_2_ measured from the right radial arterial line, and decreased central venous and/or pulmonary pressures.

Once tolerance was confirmed, clamping of the ECMO circuit was performed, followed by decannulation. Hemostasis was ensured with appropriate intervention at the cannulation sites post-decannulation.

### 2.4. Statistical Analysis

Descriptive statistics for the data were expressed as mean, standard deviation, median, minimum, maximum, frequency, and percentage values. The distribution of variables was assessed using the Kolmogorov-Smirnov test. For the analysis of quantitative independent data, the independent sample *t*-test and Mann-Whitney U test were used. For the analysis of qualitative independent data, the chi-square test was employed, and if the conditions for the chi-square test were not met, the Fisher’s exact test was applied. Statistical analyses were performed using IBM SPSS Statistics for Windows, Version 28.0 (IBM Corp., Armonk, NY, USA). A *p*-value of <0.05 was considered statistically significant.

## 3. Results

A total of 85 patients were included in the study, with 47 patients in the FC group and 38 patients in the AC group. In the FC group, there were 31 males (66%) and 16 females (34%). The mean age in this group was 56.3 ± 12.7 years. In the AC group, there were 29 males (76.3%) and 9 females (23.7%). The mean age in this group was 54.4 ± 16.2 years.

No statistically significant difference was found between the patient groups in terms of demographic data and comorbid factors (Table 1).

Intraoperative data are presented in Table 2. The majority of cardiac procedures in both groups were isolated coronary artery bypass grafting (CABG), with no significant difference in surgical type. The device implantation time was significantly shorter in the AC group compared to the FC group (26.8 ± 38.7 min vs. 59.0 ± 63.3 min; *p* = 0.015). Conversely, CC time was significantly longer in the AC group than in the FC group (93.3 ± 40.9 min vs. 70.0 ± 44.3 min; *p* = 0.029). No significant difference was observed between the groups in terms of CPB time (*p* > 0.05).

Postoperative clinical outcomes and complications are presented in Table 3 and Table 4.

There was no significant difference (*p* > 0.05) in ECMO weaning times between the FC and AC groups. The rates of postoperative vascular, neurological, and pulmonary complications were not significantly different between the FC and AC groups (*p* > 0.05). Similarly, there was no significant difference (*p* > 0.05) between the FC and AC groups in terms of postoperative drainage and revision for postoperative bleeding. The mortality rate did not differ significantly (*p* > 0.05) between the FC and AC groups.

## 4. Discussion

The ideal peripheral cannulation in VA ECMO remains a gray area. Due to its ease of application, particularly in emergency situations where it can be performed percutaneously at the bedside, femoral cannulation is frequently preferred [11]. However, as highlighted in the literature, this cannulation method can be associated with complications such as infections and extremity ischemia [12,13,14,15,16,17]. Femoral cannulation may cause differential oxygenation in patients with recovering left ventricular function and severe pulmonary dysfunction. In this situation, deoxygenated blood ejected from the native heart flows antegradely to the upper body, including the brain and coronary arteries, while oxygenated blood from the ECMO circuit flows retrogradely through the femoral artery to supply the lower body [18]. The phenomenon of differential oxygenation during VA ECMO—characterized by the coexistence of deoxygenated blood in the upper body and oxygenated blood in the lower body—has been extensively described by Falk et al., highlighting the importance of cannulation strategy and drainage configuration in minimizing hypoxemic complications [7]. As highlighted by Chicotka et al., femoral arterial cannulation may be inadequate in certain clinical scenarios due to its association with differential hypoxemia and related complications. To address these concerns, they proposed an upper-body configuration (‘sport model’) involving right internal jugular and axillary or subclavian artery cannulation to ensure more reliable and balanced oxygen delivery during VA ECMO support [6]. In contrast, axillary cannulation does not present a differential hypoxia condition. Considering all these factors, the literature suggests that right axillary cannulation is a more reliable choice for cannulation preference [19].

Significant complications related to both the patient and the device in VA ECMO patients continue to be a concern for practitioners. Studies have clearly shown the association between cannulation-related complications and survival [20]. Therefore, in this study, we investigated the effects of axillary and femoral cannulation preferences on outcomes and potential complications in patients who underwent peripheral VA ECMO.

In patients requiring VA ECMO, determining whether clinical outcomes and potential complications are related to the severity of the underlying disease or to the device itself can often be challenging. In this regard, the importance of preoperative demographic data and clinical presentation of the patients cannot be overlooked. Although the relationship between these factors and mortality and complications has not yet been fully established, several studies have reported that pre-ECMO clinical presentation is a predictor of mortality and complication rates [21,22,23,24,25,26]. In this context, preoperative demographic data and clinical presentations of patients were compared between the two groups, and no statistically significant difference was found between the groups. This indicates that the patient groups included in the study were homogeneous in terms of preoperative data. We believe that the homogeneity of preoperative data enhances the reliability of the results regarding mortality and complications, which are the endpoints of the study.

When examining the literature, it is emphasized that the overall survival of patients treated with VA ECMO in the hospital is largely dependent on the primary indication for circulatory support [27]. Studies have reported that axillary cannulation in elective, emergency, and redo cardiac surgery procedures involving CPB reduces both mortality and morbidity [28,29,30]. This is likely explained by the potential benefits of axillary cannulation. Additionally, especially in cases where VA ECMO is used for long-term support or as a bridge to permanent support, allowing time for extubation and mobilization post-surgery in patients with adequate time for axillary cannulation in the operating room is one of the reasons we tend to prefer axillary cannulation in our open-heart surgery patients.

The effects of ECMO on postoperative morbidity and mortality in open-heart surgery have been established for many years. In a study by Kalampokas et al., it was reported that 52% of patients undergoing open-heart surgery with femoral cannulation had isolated CABG, 29% had combined CABG and valve surgery, and the remaining patients had other cardiac procedures [7,31]. In a study by Pisani et al., the patients who underwent axillary cannulation were reported to have undergone heart transplantation (23%), valve surgery (12.1%), CABG (9.2%), and aortic surgery (8.6%) [32]. In our patient groups included in the study, there was no statistically significant difference in the types of cardiac surgical procedures the patients underwent. Therefore, our groups were also homogeneous in terms of intraoperative variables.

The use of ECMO to support a compromised ventricle, particularly with femoral cannulation, can lead to excessive loading of the left ventricle, causing an increase in afterload [33]. Although the optimal method for LV venting in such cases remains uncertain, the IABP is still the most commonly used support in clinical practice [34,35]. However, the combined use of ECMO and IABP remains controversial, with several studies reporting no significant benefit in terms of survival or LV unloading, and standardized clinical guidelines for ECMO application have yet to be established [36]. In a study by Ohira et al., it was reported that IABP was preferred for LV unloading in 50.3% of the femoral group and 47.7% of the axillary group [8]. In a study by Radakovic et al., IABP use was reported at 59.1% in the femoral group and 48.6% in the axillary group [37]. The most commonly used methods in our practice were IABP and atrial septostomy (3 patients). In this regard, there was no statistically significant difference in the application of IABP between the patient groups included in our study.

The potential complications of VA ECMO undoubtedly have an impact on survival outcomes [38]. Among these, the most common complications are vascular issues [20]. Studies have shown that the incidence of vascular complications in VA ECMO patients undergoing either percutaneous or surgical femoral cannulation varies between 10% and 70% [14,15,16,17]. Although groin wound infections are more common, ischemic events are the most concerning vascular complications. Additionally, studies comparing femoral and axillary cannulation have reported that vascular complications occur more frequently in the femoral group [8,39]. Surprisingly, in our study, no statistically significant difference was observed between the groups in terms of vascular-ischemic complications. This may be attributed to our routine practice of placing a distal perfusion catheter in all femoral cannulation patients and monitoring the extremity for ischemia at regular and frequent intervals. Factors such as the diameter of the distal perfusion catheter and the difficulty of performing the procedure at the bedside can certainly affect the results. It is important to recognize that technical factors such as the diameter and type of vascular graft, the method of anastomosis, and the directionality of perfusion flow may all contribute to variability in clinical outcomes. While these variables were not the primary focus of our analysis, they may have introduced heterogeneity and should be taken into account in the interpretation of the results. In our clinical practice, we minimize the technical difficulty by performing distal perfusion catheter under Color Doppler Ultrasound guidance. We believe this has positively impacted our outcomes.

In VA ECMO patients, the increase in afterload resulting from left ventricular overload is likely to lead to pulmonary congestion [33]. In these patient groups, pulmonary complications are often less addressed due to pulmonary congestion, despite the severity of the underlying condition. However, during the ECMO phase, the reality of Systemic Inflammatory Response Syndrome (SIRS) and pulmonary outcomes associated with the foreign surface is an important consideration. Furthermore, lung ischemia, which results from decreased pulsatility in bronchial circulation, is also one of the pulmonary complications [40].

From another perspective, the impaired lung function caused by VA ECMO may require prolonged mechanical ventilation (MV), which carries the risk of MV-associated pulmonary complications. In recent studies by Ohira et al., patients on peripheral VA ECMO were evaluated for extended intubation (>7 days) and tracheostomy rates. In the axillary group, extended intubation was reported in 62.4% and tracheostomy in 29.8%, while in the femoral group, the rates were 64.7% for extended intubation and 24.8% for tracheostomy [8]. In a study by Djordjevic et al., the tracheostomy rates were found to be 32% in the femoral group and 34% in the axillary group [41]. Similarly, in our study, there was no statistically significant difference between the groups. As a result, we believe that the first step in preventing pulmonary complications in these patient groups should be to provide VA ECMO support as promptly as possible. Moreover, early detection and treatment of LV overload and pulmonary congestion are considered fundamental protective factors in preventing pulmonary complications in these patients.

Neurological complications, particularly stroke, are a devastating complication of VA ECMO. Studies have reported neurological complications in up to 20% of patients [28,30]. There was no statistically significant difference in neurological complications between the patient groups in our study. This could be attributed to the fact that the etiology of neurological events is associated not only with the arterial cannulation site but also with other factors such as age, cerebrovascular disease, atrial fibrillation, left ventricular function, and the indication for VA ECMO [42,43].

Despite the increasing experience worldwide, according to the current Extracorporeal Life Support Organization (ELSO) Report, the survival rate until discharge for adults was 41.4% [44]. In a study by Wong et al., which included 103 patients, in-hospital mortality was compared between the femoral and axillary groups, and no statistically significant difference was found regarding in-hospital mortality between the groups [39]. Similarly, in our study, the mortality rates were comparable, and no significant difference in in-hospital mortality was observed between the groups.

Acute kidney injury (AKI) is one of the most common complications in ECMO patients [45]. Renal dysfunction undoubtedly affects mortality and morbidity outcomes. However, differences in renal dysfunction and its outcomes among different types of ECMO remain uncertain. In contrast to the findings reported by Ohira et al., where dialysis-requiring renal dysfunction was more frequent in the femoral cannulation group (34.8%) than in the axillary group (28.6%), our study demonstrated a higher incidence of dialysis requirement among patients who underwent axillary arterial cannulation [8]. This discrepancy may be attributed to differences in patient selection, underlying comorbidities, or institutional cannulation protocols. Additionally, a recent study evaluating combined femoral and axillary (FA+AA) cannulation reported worse preoperative renal parameters in the FA+AA group, including higher rates of chronic renal failure and elevated baseline creatinine levels, despite similar short-term mortality outcomes [46]. Taken together, these findings highlight the heterogeneity in renal outcomes associated with different cannulation strategies and suggest that patient-specific factors likely play a critical role in determining renal risk during VA-ECMO support. In our study, the rate of newly initiated dialysis was consistent with the literature, and no statistically significant difference was found between the groups in this regard.

### Study Limitations

ECMO treatment protocols, including device settings and weaning strategies, varied among patients, potentially affecting outcomes and complicating group comparisons. The lack of randomization may have introduced selection bias, limiting comparability between axillary and femoral ECMO groups. Additional limitations include the study’s retrospective nature, single-center design, small sample size, and limited outpatient follow-up—common in ECMO populations due to high mortality and post-discharge challenges—potentially reducing statistical power. Technical factors such as cannula size, graft diameter, method of anastomosis, and directionality of perfusion flow may have affected outcomes and introduced heterogeneity. These aspects were not the primary focus of our analysis but should be considered when interpreting the results.

## 5. Conclusions

Achieving safe arterial cannulation for VA ECMO is crucial, as complications in this patient group can affect the entire treatment management and, consequently, survival. Utilizing two distinct peripheral arterial cannulation sites offers a flexible and patient-tailored approach. This allows for a more individualized decision-making process regarding the choice of cannulation method. In our study, the results in both the axillary and femoral cannulation groups were consistent with the literature. When tailored to patient-specific characteristics, both approaches may be considered feasible and potentially safe. However, randomized controlled trials with an equivalence are needed to determine the optimal cannulation strategy.

## Figures and Tables

**Table 1:** Patient’s Demographics and Clinical Features.

	FC (n = 47)		AC (n = 38)		
	Mean ± SD	n (%)	Mean ± SD	n (%)	*p*-Value
Age (years)	56.3 ± 12.7		55.4 ± 16.2		0.891
Gender					
Female		16 (34.0%)		9 (23.7%)	0.297
Male		31 (66.0%)		29 (76.3%)	
BSA (m^2^)	1.8 ± 0.2		1.8 ± 0.2		0.761
EuroSCORE II	7.5 ± 8.5		7.5 ± 6.8		0.372
Comorbidity					
DM		18 (38.3%)		16 (42.1%)	0.722
HT		27 (57.4%)		17 (44.7%)	0.244
COPD		11 (23.4%)		7 (18.4%)	0.576
HLP		12 (25.5%)		9 (23.7%)	0.844
PAD		2 (4.3%)		2 (5.3%)	1.000
CRF		5 (10.6%)		4 (10.5%)	0.987
Smoking		27 (57.4%)		18 (47.4%)	0.355
MI		30 (63.8%)		17 (44.7%)	0.078
Use of IABP		26 (55.3%)		23 (60.5%)	0.629

BSA: body surface area; DM: diabetes mellitus; HT: hypertension; COPD: chronic obstructive pulmonary disease; HLP: hyperlipidemia; PAD: peripheral arterial disease; CRF: chronic renal failure; MI: myocardial infarction; IABP: intra-aortic balloon pump; FC: femoral cannulation; AC: axillary cannulation.

**Table 2:** Intraoperative Data.

	FC (n = 47)		AC (n = 38)		
	Mean ± SD	n (%)	Mean ± SD	n (%)	*p*-Value
Surgical Procedure					
CABG		24 (51.0%)		20 (52.6%)	0.849
MVR		10 (21.2%)		6 (15.7%)	0.115
AVR		12 (25.5%)		11 (28.9%)	0.551
TVR		1 (2.1%)		1 (2.6%)	1.000
CC Time (min)	70.0 ± 44.3		93.3 ± 40.9		0.029
CPB Time (min)	135.9 ± 89.8		175.6 ± 78.3		0.089
ECMO Implantation Time (hour)	59.0 ± 63.3		26.8 ± 38.7		0.015
IABP Use After ECMO		7 (14.9%)		6 (15.8%)	0.909

CABG: coronary artery bypass grafting; MVR: mitral valve replacement; AVR: aortic valve replacement; TVR: tricuspid valve replacement; CC: cross-clamp; CPB: cardiopulmonary bypass; ECMO: extracorporeal membrane oxygenation.

**Table 3:** Postoperative Clinical Outcomes.

	FC (n = 47)		AC (n = 38)		
	Mean ± SD	n (%)	Mean ± SD	n (%)	*p*-Value
Prolonged Intubation (>24 h)		41 (87.2%)		31 (81.6%)	0.471
Extubation Time (hour)	65.9 ± 58.2		62.4 ± 71.3		0.681
Time on ECMO (day)	6.9 ± 3.5		6.6 ± 8.7		0.913
Successful Weaning from ECMO		21 (44.6%)		15 (39.4%)	0.629
Surgical Exploration for Decannulation		12 (25.5%)		4 (10.5%)	0.078
ICU Stay (day)	15.3 ± 15.5		10.9 ± 11.9		0.139
Discharged		16 (34.0%)		9 (23.7%)	0.297
Total Hospital Stay (day)	57.6 ± 44.8		47.6 ± 29.9		0.853
Mortality		32 (68.1%)		29 (76.3%)	0.402

ECMO: extracorporeal membrane oxygenation; ICU: intensive care unit.

**Table 4:** Postoperative Complications and Interventions.

	FC (n = 47)		AC (n = 38)		
	Mean ± SD	n (%)	Mean ± SD	n (%)	*p*-Value
Complications					
Vascular Complications		4 (8.5%)		1 (2.6%)	0.252
Neurological Complications		9 (19.1%)		9 (23.7%)	0.611
Pneumonia		12 (25.5%)		9 (23.7%)	0.844
Tracheostomy		1 (2.1%)		2 (5.3%)	0.584
Postoperative Drainage (mL)	1183.0 ± 1744.8		2035.0 ± 3454.7		0.077
Bleeding Requiring Surgical Revision		13 (27.7%)		17 (44.7%)	0.101
Dialysis Requirement		12 (25.5%)		14 (36.8%)	0.261
Blood Transfusion (unit)	9.9 ± 10.1		8.9 ± 7.5		0.958

## Data Availability

The data presented in this study are available on request from the corresponding author due to ethical reasons.
